# Supplementary material for: Rapamycin Exerts Its Geroprotective Effects in the Ageing Human Immune System by Enhancing Resilience Against DNA Damage
Source: Aging Cell. 2026 Jan 12;25(2):e70364. doi: 10.1111/acel.70364 (PMC12794675; doi:10.1111/acel.70364)
Supplement: Supplementary file 1 — Figure S1: Gating strategy for CD4+ and CD8+ T cells using conventional flow cytometry after in vitro T‐cell‐specific activation of PBMCs. Figure S2: Effects of mTOR inhibitors on human T cell activation over 3 days. Figure S3: Mitigation of DNA damage markers by rapamycin is not due to modulation of the cell cycle or protein synthesis. Figure S4: Flow cytometry‐based measurement of autophagic flux. Figure S5: Autophagy is required for the resolution of DNA damage, but is not required for the mitigation of DDR upregulation by rapamycin. Figure S6: Gating strategy for PBMCs using 27‐colour spectral flow cytometry. Figure S7: In vivo rapamycin treatment in older humans. Table S1: Details of drugs used in cell culture experiments. [file ACEL-25-e70364-s002.pdf]

SUPPLEMENTARY MATERIAL

CONTENTS

Item	Title	Page
Figure S1	Gating strategy for $CD4^+$ and $CD8^+$ T cells using conventional flow cytometry after *in vitro* T-cell-specific activation of PBMCs	2
Figure S2	Effects of mTOR inhibitors on human T cell activation over 3 days	3
Figure S3	Mitigation of DNA damage markers by rapamycin is not due to modulation of the cell cycle or protein synthesis	4
Figure S4	Flow cytometry-based measurement of autophagic flux	5
Figure S5	Autophagy is required for the resolution of DNA damage, but is not required for the mitigation of DDR upregulation by rapamycin	6
Figure S6	Gating strategy for PBMCs using 27-colour spectral flow cytometry	7
Figure S7	*In vivo* rapamycin treatment in older humans	8
Table S1	Details of drugs used in cell culture experiments	9
Table S2	Details of antibodies used in the study	Separate file

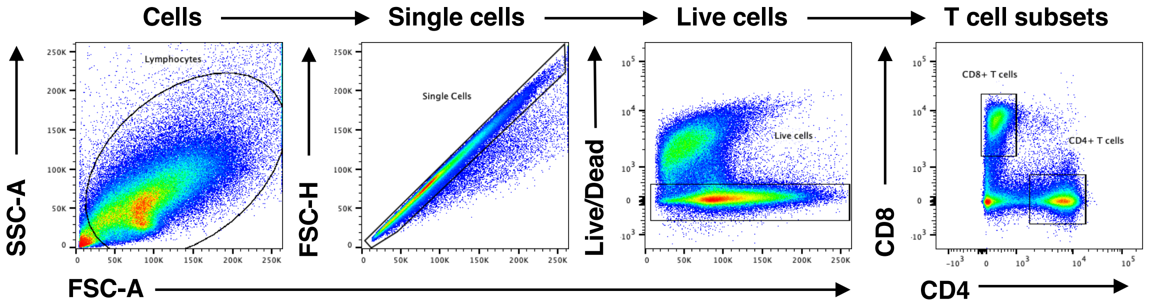

Figure S1 Gating strategy for CD4+ and CD8+ T cells using conventional flow cytometry after in vitro T-cell-specific activation of PBMCs

Total cells are gated based on size (forward scatter, FSC-A) and granularity (side scatter, SSC-A) to remove debris. Single cells are determined by their direct proportionality between FSC-area (FSC-A) and FSC-height (FSC-H). Live cells are identified by their absence of fluorescence of a membrane-permeable dye. T cell subsets are then identified by their high expression of either CD4 or CD8.

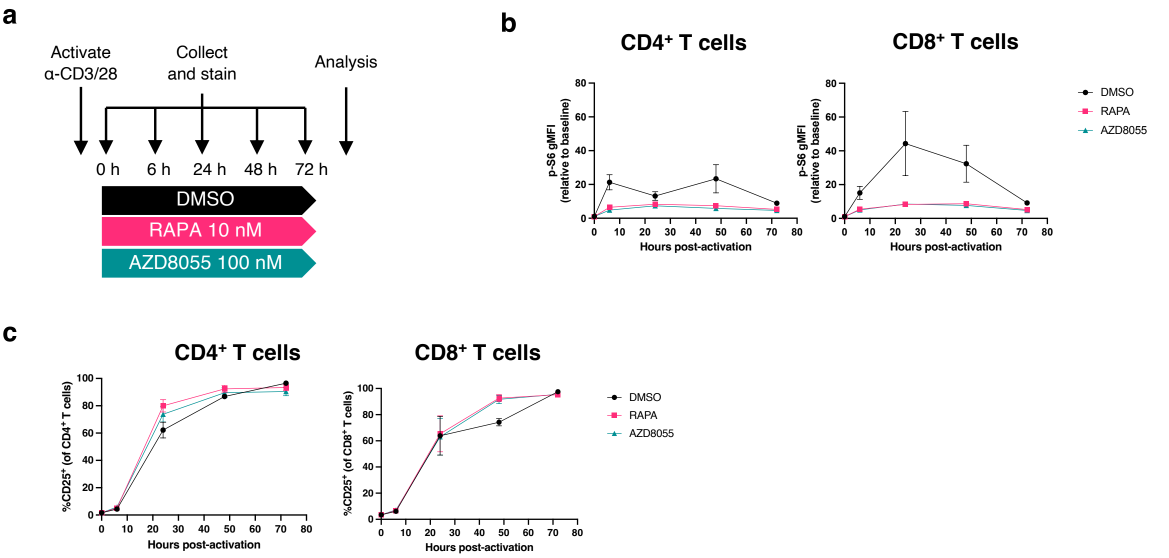

Figure S2 Effects of mTOR inhibitors on human T cell activation over 3 days

(a) Experimental design for 3-day T-cell-specific activation of PBMCs from healthy donors with 1 µg/ml α-CD3/28 each, in the presence of 10 nM rapamycin (RAPA), 100 nM AZD8055, or DMSO control, with analysis by flow cytometry. (b) p-S6 geometric mean fluorescence intensity (gMFI) in total $CD4^+$ (left) and $CD8^+$ (right) T cells relative to baseline. (c) Proportion of cells positive for CD25 across the 3-day activation in flow cytometry-gated $CD4^+$ or $CD8^+$ T cells.

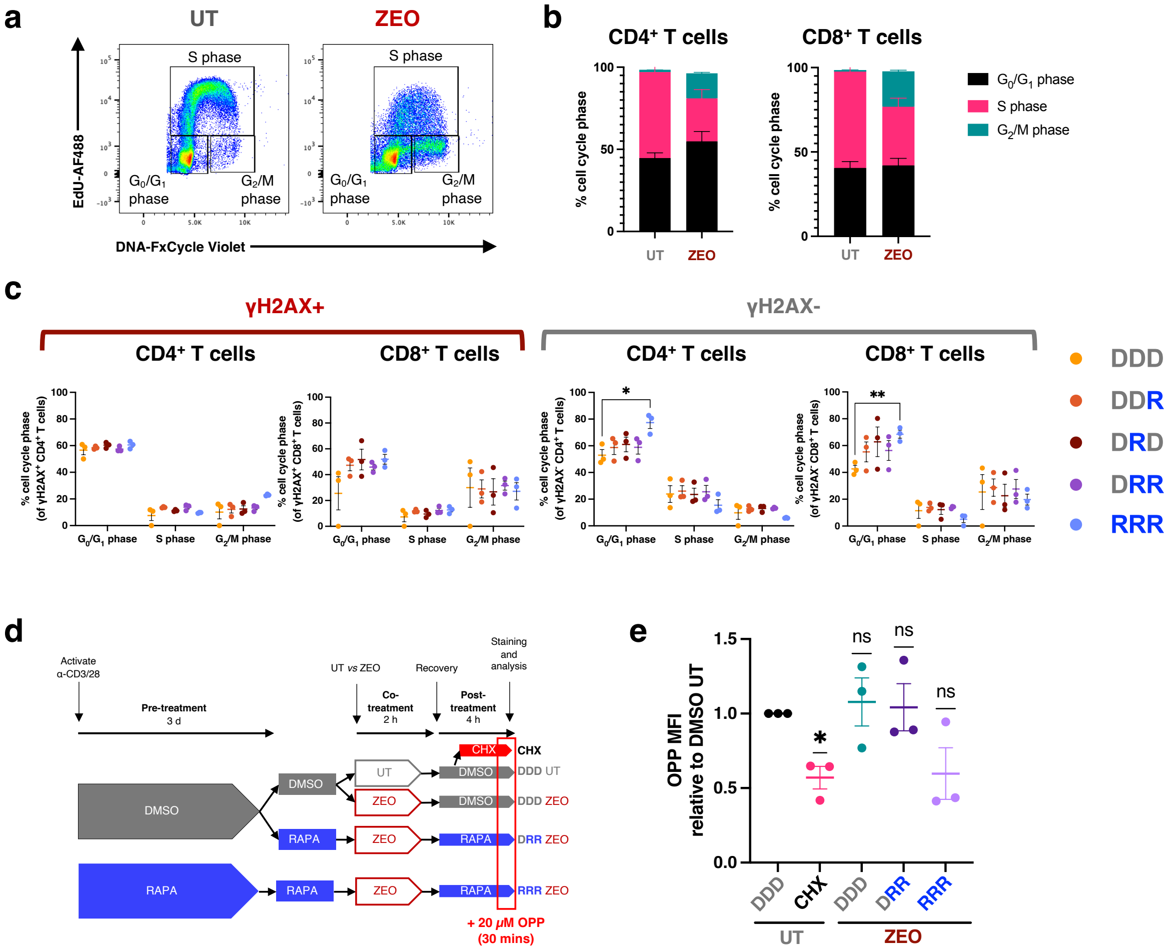

Figure S3 Mitigation of DNA damage markers by rapamycin is not due to modulation of the cell cycle or protein synthesis

(a-b) Representative histograms for cell cycle phases in untreated (UT) and zeocin-treated conditions with quantification in (b), n=3 healthy donors. (c) Cell cycle phases in zeocin-treated T cells treated with rapamycin continuously ("RRR"), during ("DRD"), and/or after ("DRR", "DDR") zeocin exposure, gated as positive or negative for γH2AX, n=3 healthy donors. Unless otherwise indicated, statistical comparisons between different treatment conditions within each cell cycle phase (G_0/G_1, S, G_2/M) were not significant, as determined by a two-way ANOVA with Dunnett's multiple comparisons test. (d) Experimental design for measuring nascent protein synthesis with 20 μM O-propargyl-puromycin incorporation over 30 minutes. 1-hour treatment with 50 μg/ml cycloheximide (CHX) was used a positive control for inhibition of protein synthesis. (e) OPP mean fluorescence intensity (MFI) of CD4+ T cells, relative the DMSO untreated (UT) control, across conditions undergoing treatment with 10 nM rapamycin continuously ("RRR") or during and after zeocin exposure ("DRR"). *P*-values are derived from a one-sample t-test (theoretical mean = 1), n=3 donors.

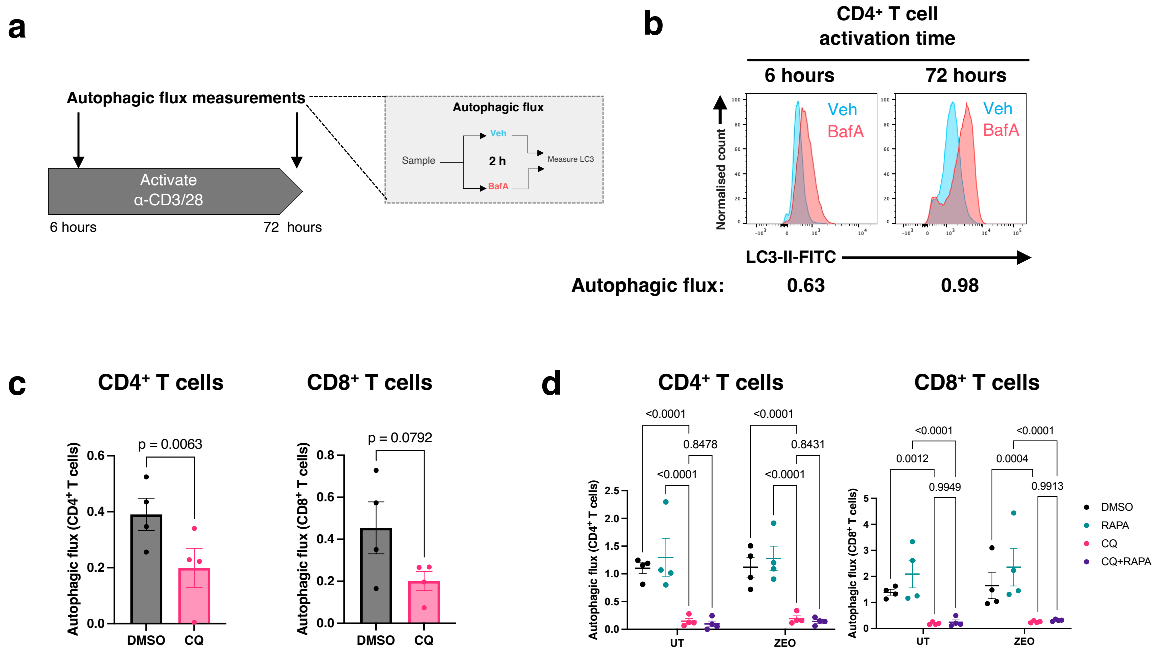

Figure S4 Flow cytometry-based measurement of autophagic flux

(a) Experimental design in which PBMCs from healthy donors underwent T-cell-specific activation with 1 μg/ml α-CD3/28. At 6 hours and 72 hours of activation, cells were retrieved and treated with either 10 nM bafilomycin A_1 or DMSO vehicle control (Veh) as indicated, and autophagic flux measured using a flow cytometry-based LC3 assay. (b) Representative fluorescence histograms of LC3 levels in gated CD4+ T cells after 6 and 72 hours of activation as in (a), with autophagic flux indicated below. Representative of 3 independent experiments. (c-d) Autophagic flux in CD4+ and CD8+ T cells undergoing 3-day activation in the presence of (c) chloroquine (CQ, 10 μM) or DMSO vehicle control or (d) following zeocin treatment (or untreated) after 3-day activation in chloroquine (CQ, 10 μM), rapamycin (RAPA, 10 nM), or both (CQ+RAPA), n=4 healthy donors. P-values are derived from a paired t-test (c) or two-way ANOVA with Tukey's multiple comparisons test (d).

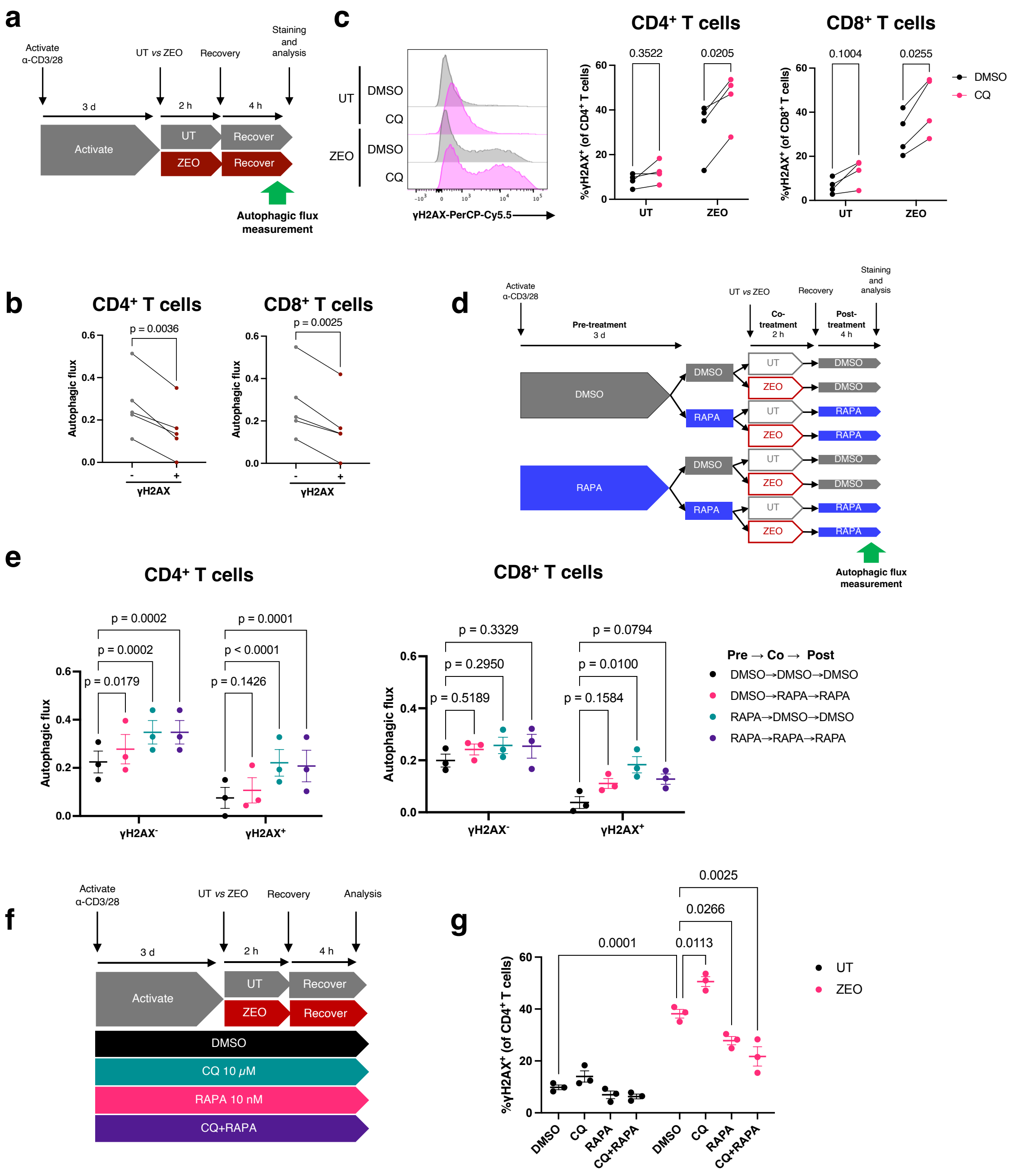

Figure S5 Autophagy is required for the resolution of DNA damage, but is not required for the mitigation of DDR upregulation by rapamycin

(a) Experimental design for combined DNA damage and autophagic flux assay. (b) Quantification of autophagic flux in γH2AX^{+} and γH2AX^{-} populations in flow cytometry-gated CD4^{+} and CD8^{+} T cells, n=5 healthy donors. (c) Representative γH2AX histograms (left) and quantified percentage (right) of γH2AX^{+} CD4^{+} and CD8^{+} T cells after zeocin treatment following 3-day T cell-specific activation in chloroquine (CQ, 10 μM) or DMSO control, n=4 healthy donors. (d) Experimental design for measuring autophagic flux in CD4^{+} and CD8^{+} T cells across different rapamycin treatment conditions with respect to zeocin exposure. (e) Autophagic flux in zeocin-exposed cells gated as positive or negative for γH2AX, across rapamycin treatment conditions. (f) Experimental design for treatment of zeocin-exposed activated T cells with chloroquine (CQ), rapamycin (RAPA), or dual treatment (CQ+RAPA). (g) Percentage of γH2AX^{+} CD4^{+} T cells after zeocin treatment across conditions in (f), n=3 healthy donors. P-values are derived from a paired t-test (b), a two-way ANOVA with Šídák's (d,e) or Tukey's multiple comparisons test (g).

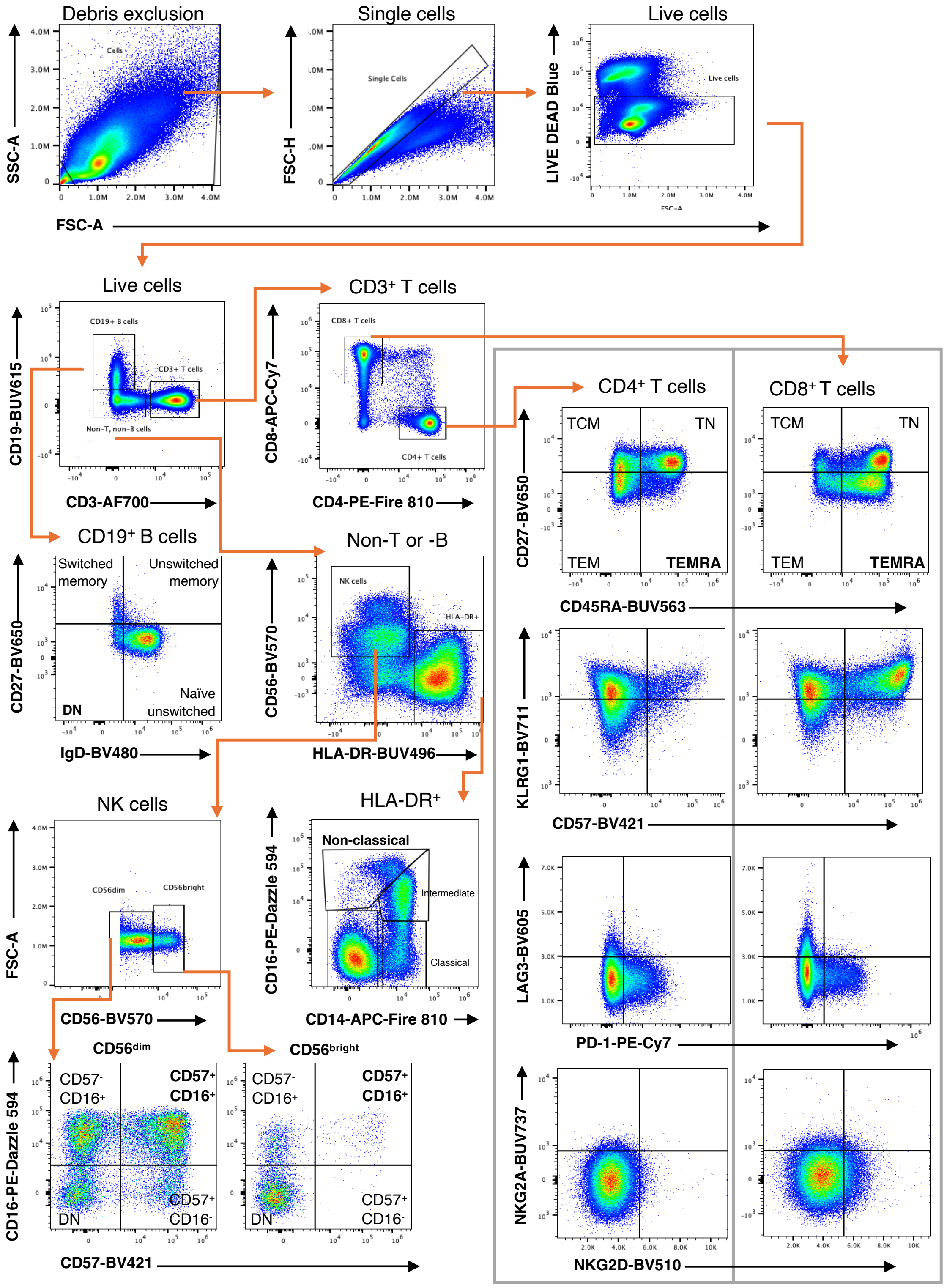

Figure S6 Gating strategy for PBMCs using 27-colour spectral flow cytometry

Gates are indicated. Orange arrows indicate where a population has been further gated upon.

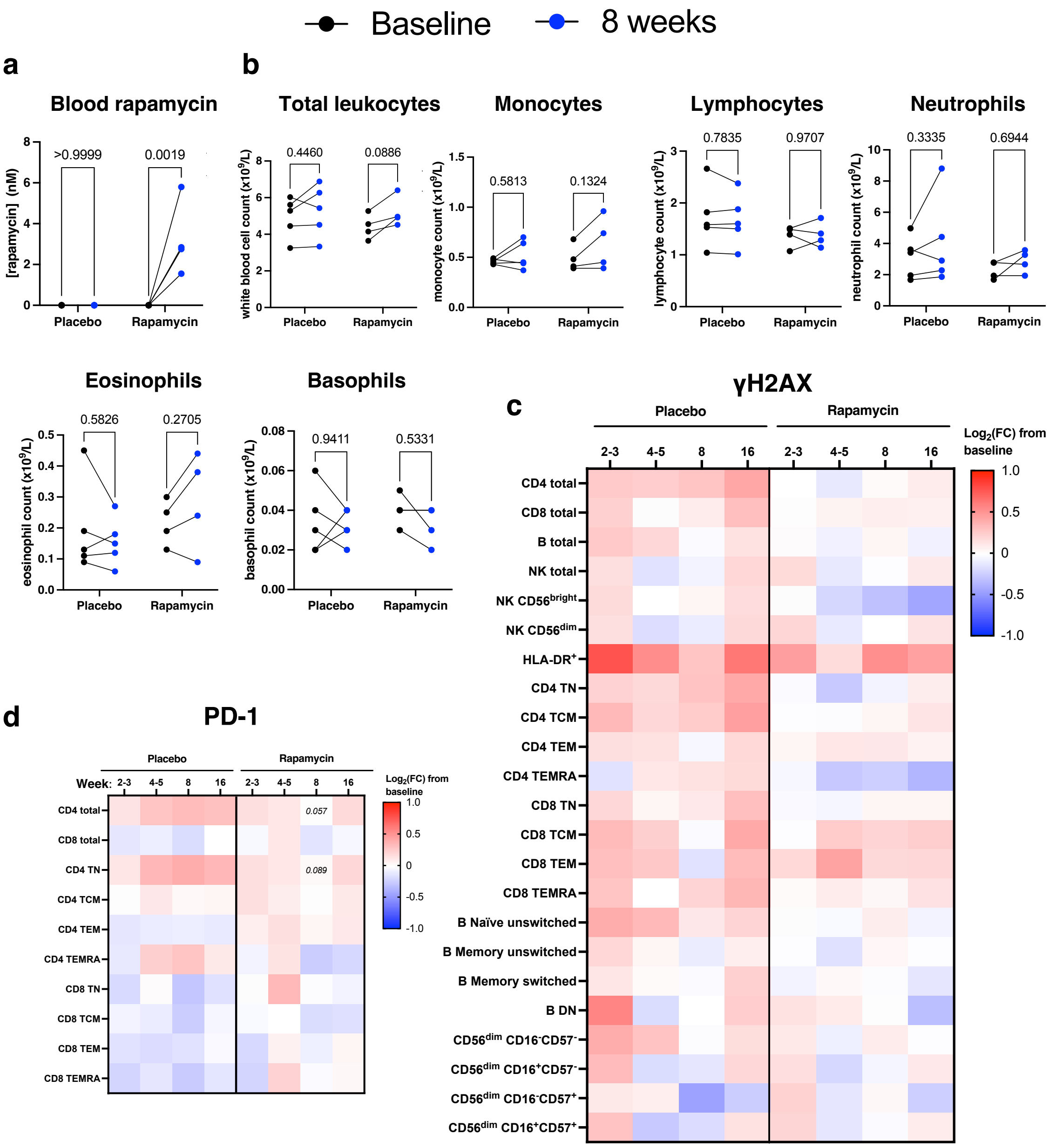

Figure S7 ***In vivo*** **rapamycin treatment in older humans**

(a-b) (a) Blood concentration of rapamycin and (b) total white blood cells, monocytes, lymphocytes, neutrophils, eosinophils, and basophils in participants of placebo and rapamycin groups at week 8 of the study (n=5 placebo, n=4 rapamycin). (c) γH2AX geometric mean fluorescence intensity across immune subsets in the rapamycin trial. (d) Proportion of T cells positive for PD-1 across defined T cell subsets in participants in rapamycin (n=4) and placebo (n=5) groups. In (c-d), each value is expressed as $\log_2$(fold change) from baseline for each participant. Statistical tests in (c-d) are derived from an unpaired t-test between placebo and rapamycin at each time point. *P*-values in (a-b) are determined by two-way ANOVA with Šídák's multiple comparisons tests.

Table S1 Details of drugs used in cell culture experiments

Name	Target	Final concentration	Solvent	Manufacturer	Cat. code
Zeocin	DSB inducer	200 μg/ml	H_2O	Invitrogen	R25001
Rapamycin	mTORC1 inhibitor	10 nM	DMSO	Alfa Aesar	J67452
AZD8055	Pan-mTOR inhibitor	100 nM	DMSO	Stratech Scientific	A8214-APE-10mM
Bafilomycin A_1	Autophagy inhibitor	10 nM	DMSO	Sigma	B1793-10UG
Hydrogen peroxide	Oxidative stress	25 μM	PBS	Sigma	H1009-100ML
Chloroquine	Autophagy inhibitor	10 μM	DMSO	Sigma	C6628-25G
Cycloheximide	Protein translation inhibitor	50 μg/ml	DMSO	Sigma	239763-M

N.B. DMSO vehicle controls always contained 0.1% DMSO (v/v) in the cell culture media.

Table S2 Details of antibodies used in the study – see separate file.
